# Post-bariatric hypoglycaemia: from altered gut physiology to targeted therapies

**DOI:** 10.1530/EC-25-0850

**Published:** 2026-08-05

**Authors:** Wei Yang, Tricia M-M Tan

**Affiliations:** Section of Endocrinology and Investigative Medicine, Department of Metabolism, Digestion and Reproduction, Faculty of Medicine, Imperial College London, London, UK

**Keywords:** obesity, bariatric surgery, post-bariatric hypoglycaemia, physiology, mechanism, treatment

## Abstract

Post-bariatric hypoglycaemia (PBH) is an increasingly recognised complication of bariatric and metabolic surgery, which can markedly impair quality of life and jeopardise the long-term benefits of surgery. This review summarises the current evidence on the epidemiology, pathophysiology, and management of PBH, with a particular focus on mechanism-based treatment strategies. We first point out the limitations of existing epidemiological data and then outline a practical diagnostic framework. We next review the evolving understanding of PBH as a heterogeneous, pathophysiology-driven syndrome arising from rapid nutrient delivery, exaggerated incretin responses, excessive insulin secretion, impaired glucagon secretion, and emerging contributors, such as postprandial serotonin hypersecretion, bile acid signalling, and microbiome change. On this basis, we review therapeutic options by mechanistic target, covering dietary and lifestyle interventions, acarbose, SGLT inhibition, somatostatin analogues, GLP-1 receptor antagonists, diazoxide, glucagon-based approaches, and experimental serotonin receptor antagonism, and briefly discuss agents with limited supportive evidence. Finally, we explore key knowledge gaps and future directions, including the need for long-term prospective data, precision medicine approaches, rational combination therapy, and greater integration of continuous glucose monitoring and digital decision support. Mechanism-based, multidisciplinary care from bariatric physicians, dietitians, and surgeons offers the best prospect of managing PBH, preserving quality of life, and safeguarding the metabolic benefits of bariatric surgery.

## Introduction

Bariatric surgery has revolutionised the treatment of morbid obesity and its complications. Interventions such as Roux-en-Y gastric bypass (RYGB) and sleeve gastrectomy (SG) lead to a sustained weight loss and are associated with favourable changes in obesity-related comorbidities, such as remission of type 2 diabetes (1). They lower mortality and are used increasingly often, not only for weight-loss but also as a metabolic surgery.

Even with the best results presented, surgery is not risk-free. One of its particularly difficult-to-manage complications is post-bariatric hypoglycaemia (PBH), also known as late dumping syndrome. PBH is characterised by repeated, frequently severe, postprandial hypoglycaemia, occasionally several years after surgery, and has a significant impact on daily life. Patients often complain of adrenergic symptoms (such as sweating, tremor, and palpitation), which may evolve into neuroglycopaenia as seizures, confusion, and impaired consciousness. In severe cases, PBH is associated with accidents, restricted employment opportunities and life quality, and restrictions to daily activities such as driving (2).

The pathogenesis of PBH centres on rapid nutrient delivery to the small intestine, an exaggerated incretin response, and an excessive insulin secretion culminating in postprandial glucose overswing. PBH is a heterogeneous entity, indicating a multifactorial pathophysiology (3). Clinically, PBH is a condition that is frequently overlooked. Patients are often diagnosed late (4).

Although dietary modification remains the mainstay of therapy, a subset of patients requires pharmacological or surgical interventions to achieve adequate glycaemic control (2, 3, 4). The absence of a global consensus on diagnostic and management pathways underscores the need for a deeper understanding of PBH’s epidemiology, pathophysiology, and therapeutic landscape to inform evidence-based, mechanism-driven care.

## Epidemiology

Epidemiology estimates vary because definitions and diagnostic thresholds differ. Early registry-based studies reported PBH prevalence of only 0.1–0.9%, but these figures are likely underestimates because registries mainly capture severe, clinically recognised cases (5). In contrast, observational studies revealed 20–30% of RYGB patients reporting symptoms (1, 6). Provocative tests, such as mixed meal tests, detect biochemical hypoglycaemia in 10–40% (7, 8), and continuous glucose monitoring (CGM) in free living settings can detect hypoglycaemia in up to 75% (5, 9, 10), although it is unclear what the clinical relevance of CGM-detected hypoglycaemia in the absence of symptoms is.

The risk factors for PBH may include younger age, female sex, greater postoperative weight loss, absence of preoperative diabetes, lower baseline HbA1c, higher β-cell function, prior cholecystectomy, and use of SSRIs/SNRIs (2, 3, 4, 11). Above all, the most important factor is the procedure that has been undertaken. Severe PBH is more frequent after RYGB than after SG, although both procedures can cause PBH. It is uncommon with adjustable gastric banding. Biliopancreatic diversion and one-anastomosis gastric bypass can also lead to PBH, but data are limited (2, 3, 12).

## Diagnosis

PBH is hard to diagnose due to variable symptomatology, absence of standardised tests, and overlap with other post-surgical syndromes. UK guidance recommends a pragmatic approach (2). PBH should be suspected in patients who have had bariatric surgery at least one year before, and hypoglycaemic symptoms are usually reported between 1 and 4 h after eating. Whipple’s triad should be fulfilled: i) hypoglycaemic symptoms (adrenergic and/or neuroglycopaenic), ii) biochemical measurement of blood glucose < 3.0 mmol/L (capillary values are acceptable if they are taken using a testing system which conforms to ISO 15197), and iii) symptom resolution after glucose correction.

PBH should also be differentiated from early dumping (<2 h post-meal with GI/vasomotor symptoms) and other causes, such as insulinoma, non-insulinoma pancreatogenous hypoglycaemia, adrenal insufficiency, severe liver disease, malnutrition, and drug-induced hypoglycaemia (2, 13, 14).

Dynamic testing is not generally helpful for PBH diagnosis. OGTT is poorly tolerated and nonspecific, as many normal people without upper GI or bariatric surgery can experience hypoglycaemia after an OGTT. MMTT is more physiological but unstandardised and is also not recommended for diagnosis. Symptom questionnaires are not well validated (2, 7, 8, 11).

CGM can be a useful adjunct to characterise glycaemic patterns in daily life (timing, frequency, and nocturnal and asymptomatic events) and to relate symptoms to glucose trends, but it is not currently recommended as a stand-alone diagnostic test for PBH (2). CGM may report ‘false lows’, particularly in the hypoglycaemic range, and readings can be affected by sensor lag and compression artefact. Therefore, confirmatory capillary testing is advisable when CGM values are discordant with symptoms or when documenting clinically significant hypoglycaemia.

Beyond assessment, CGM may also function as a practical intervention: real-time CGM with alerts can support behavioural modification (e.g. meal composition/spacing), earlier treatment of impending hypoglycaemia, and improved confidence, particularly in those with impaired awareness. In a masked-to-unmasked CGM study in PBH, access to CGM data and alarms was associated with reductions in hypoglycaemia and glycaemic variability (15). More recently, a randomised crossover trial reported that unblinded CGM reduced the frequency/severity of hypoglycaemia and improved hypoglycaemia-related quality of life, enabling a less restrictive diet (16).

Hence, PBH is defined pragmatically on history as i) biochemically confirmed hypoglycaemia < 3.0 mmol/L (54 mg/dL), ii) bariatric surgery occurring > 1 year ago, and iii) hypoglycaemic episodes fulfilling Whipple’s triad (see above). Alternative causes of hypoglycaemia are excluded.

## Evidence base and limitations

Clinical evidence for PBH therapies remains limited. Most studies are small and short, often use crossover designs within the same individuals, and frequently lack placebo comparators. Primary outcomes also vary (e.g. symptom scores, mixed meal glucose nadir, or CGM-derived time below range), which complicates comparison across interventions and settings (2, 3, 4, 17).

In this review, we use the term ‘long term’ to indicate follow-up beyond approximately 3 months (and ideally ≥6–12 months), as PBH is chronic and treatment durability and safety cannot be assessed over days to a few weeks (2, 3, 4).

Only a minority of pharmacological therapies have been evaluated in placebo-controlled studies with repeat dosing beyond 1–2 weeks. Examples include a double-blind placebo-controlled crossover trial of once-daily empagliflozin over 20 days (18) and outpatient studies of ready-to-use glucagon analogues, such as dasiglucagon, over several weeks (19). For many other agents, the literature is restricted to single-meal physiological studies, case series, or brief open-label trials (2, 3, 4, 17).

Several commonly used PBH agents (e.g. acarbose and SGLT inhibitors) were originally developed to lower glucose in diabetes. In PBH, their rationale is to blunt early postprandial hyperglycaemic peaks that drive downstream incretin/insulin secretion and subsequent nadirs. However, because these agents modify glucose handling, they can theoretically worsen or prolong hypoglycaemia in susceptible individuals, particularly when combined, highlighting the importance of cautious titration, CGM-supported monitoring, and larger placebo-controlled trials (2, 3, 4, 18, 20).

Given this evidence landscape, mechanistic ‘personalisation’ is appealing but can be artificial: several drugs act on multiple steps in the postprandial cascade (e.g. slowing carbohydrate absorption and thereby indirectly reducing incretin-driven insulin secretion).

## Pathophysiology and treatment options

PBH is now recognised as a heterogeneous syndrome arising from altered gastrointestinal anatomy and physiology. For clarity, we organise treatment options by predominant pathophysiological mechanisms and targets; however, these groupings are intended as a pragmatic organising framework rather than a strict classification, as many interventions act across overlapping pathways. For example, strategies that slow intestinal glucose appearance (e.g. dietary modification, acarbose, and SGLT inhibition) may also reduce downstream GLP-1 and insulin secretion. To emphasise the fact that treatments exert their effects via multiple likely mechanisms, Table 1 summarises the principal and secondary physiological targets of each intervention and the current level of supporting evidence. While personalised selection of therapy is appealing, this remains constrained by the limited size and duration of available studies, underscoring the need for larger, longer, well-controlled trials.

**Table 1:** Overlapping targets and key evidence gaps.

Intervention	Primary physiological targets (overlapping)	Best available clinical evidence (PBH-specific)	Typical study duration	Key practical considerations/limitations
Dietary/lifestyle (including meal patterning)	Slows nutrient delivery; reduces rapid glucose absorption; attenuates GLP-1/insulin peaks	Guideline/consensus; observational experience; limited controlled studies	Weeks–months (variable)	Cornerstone; adherence limiting; does not address all severe PBH
CGM	Detection of patterns; supports behaviour change; alerts and decision support	Masked-to-unmasked CGM study and randomised crossover trial showing reduced hypoglycaemia and improved QoL (15, 16)	Weeks–months	Not diagnostic alone; fingerstick/venous confirmation needed; false lows in rapid change
Acarbose (α-glucosidase inhibitor)	Delays carbohydrate digestion/absorption → lower postprandial peaks and downstream insulin/GLP-1	Small trials/case series; short crossover comparisons within individuals	Single test days to few weeks	GI side effects common; titration; may be combined cautiously with other agents
SGLT inhibition (SGLT-2 and/or SGLT-1/2)	Reduces postprandial glucose excursion and insulin demand; may blunt GLP-1 indirectly	Randomised double-blind placebo-controlled crossover trial: empagliflozin 25 mg once daily (18); plus small pilot/case reports	∼20 days (18); short pilots	Risk: volume depletion, genitourinary infections; counsel hydration; monitor for worsening hypoglycaemia especially with combination therapy
Somatostatin analogues (octreotide, pasireotide, and lanreotide)	Suppresses gut hormone release and insulin; slows gastric/intestinal transit	Mostly short-term physiologic studies; limited head-to-head data; crossover comparisons in small cohorts (17, 46)	Single doses to weeks	Injection burden; GI side effects; risk of hyperglycaemia and gallstones; specialist use
GLP-1 receptor antagonists (e.g. avexitide)	Blocks exaggerated GLP-1 signalling → reduces insulin secretion	Phase 2 trials including repeated dosing and crossover designs (60); ongoing larger trials (44, 45)	∼2–4 weeks in available trials	Promising mechanism-directed therapy; access limited; longer-term outcomes awaited
Insulin secretion modifiers (diazoxide and CCBs)	Reduces β-cell insulin secretion via K-ATP opening or Ca2+ channel blockade	Case reports/series; occasional short comparative studies (17)	Days–weeks	Off-label; side effects limit use; careful selection and monitoring
Glucagon-based rescue/automated delivery	Raises glucose via hepatic output	Proof-of-concept studies; outpatient dasiglucagon trials (19)	Single visits to weeks	May reduce severe episodes; device access; does not address root cause
Surgical management (selected cases)	Restores/adjusts anatomy to slow nutrient transit and absorption	Retrospective series; highly selected patients (31)	Months–years (observational)	Invasive; reserved for refractory PBH

CGM, continuous glucose monitoring; CCBs, calcium channel blockers.

### Mechanism 1: rapid nutritional/carbohydrate absorption

Following bariatric procedures, particularly RYGB, the normal process of gastric emptying and nutrient absorption is profoundly altered. In RYGB, ingested food rapidly bypasses the pylorus and proximal small intestine, reaching the jejunum within minutes and eliminating the controlled, gradual nutrient delivery normally mediated by the pylorus. This accelerated nutrient transit triggers a rapid rise in plasma glucose followed by an exaggerated incretin response (GLP-1 and GIP) and insulin secretion. Although SG preserves pyloric function, reduced gastric compliance and altered antral motility can still accelerate gastric emptying and small-intestinal nutrient delivery, especially for liquids, contributing to earlier glycaemic and hormonal peaks (21). The resulting mismatch between early insulin secretion and subsequent glucose appearance contributes to the rapid glucose decline characteristic of PBH.

#### Treatment options for mechanism 1

##### Dietary and lifestyle management

Diet and lifestyle modifications are generally agreed to be the first-line and most reliable intervention for PBH. By slowing gastric emptying and intestinal nutrient delivery, they dampen the rapid nutrient absorption (especially of carbohydrates) that drives glycaemic swings. People living with PBH should be informed that nutritional/dietary therapy is a medical treatment and should be educated, according to ability, on the rationale and principles for the dietary and behavioural changes (11). In addition, they should have a full dietetic review by a registered dietitian, preferably with a specialist interest in bariatric surgery. This is essential because standard nutritional surveillance may reveal deficiencies in iron, vitamin B12, folate, vitamin D, calcium, and trace elements, all of which can exacerbate fatigue and neurological symptoms that may be misinterpreted as hypoglycaemia (2, 22). Meeting protein and micronutrient needs provides a safe foundation before more specific strategies are applied.

##### Control of carbohydrate intake

The single most important dietary principle is controlling the quality and quantity of carbohydrate. Eating smaller, more frequent meals (5–6 times a day) with <30 g carbohydrate per meal may be helpful (23). Patients should also be encouraged to shift their consumption to low glycaemic index (GI) carbohydrates (for example, whole grains, and non-starchy vegetables) to help slow down digestion and absorption (2). Clinical studies show that replacement of high-GI foods by low-GI products is both feasible and effective in lowering postprandial glucose variability (3).

##### Increased protein intake

Adequate protein is generally recommended after bariatric surgery and is usually prescribed relative to body weight (for example ∼1.0–1.5 g/kg/day based on ideal or adjusted body weight, individualised to procedure type and nutritional status), rather than a single fixed threshold. In PBH, prioritising protein consumption with each meal or snack can help reduce the relative carbohydrate load and may attenuate rapid postprandial glycaemic excursions (2, 22).

##### Role of dietary fat and fibre

Dietary fat and soluble fibre both suppress gastric and intestinal transit of food, resulting in a flatter glycaemic profile. Small studies with fat- or fibre-enriched meals show decreased frequency of postprandial hypoglycaemia (3). Patients should be counselled on eating healthy unsaturated plant-derived fats (for example, olive or rapeseed oil and avocadoes) and fibre. However, a balance is required as too much fat consumption (of any type) may predispose to weight gain.

##### Abstinence from fluid intake around mealtimes

Solid meals are absorbed more slowly and are associated with a lower incidence of hypoglycaemia and a reduction in peak GLP-1 response to feeding in comparison with liquid meals (24). Patients with PBH should therefore be advised to abstain from fluids from 30 min before each meal to 30 min after (2).

##### Abstinence from alcohol

Alcohol avoidance is important. Many alcoholic drinks contain quickly absorbed carbohydrates, which would provoke PBH. Alcohol inhibits hepatic gluconeogenesis, predisposing towards a prolonged hypoglycaemia. In addition, the risk of alcohol use disorder is increased following bariatric surgery (2, 4).

##### Utility of continuous glucose monitoring (CGM)

Although CGM is not recommended as a stand-alone diagnostic tool, it is a rational and widely used adjunct for day-to-day management. CGM can characterise the timing, frequency, and severity of postprandial glucose dips, identify asymptomatic events, and support iterative dietary adjustment (e.g. meal composition, spacing, and rescue strategies) (2, 7, 9).

From a practical perspective, CGM may also reduce fear of hypoglycaemia and improve patients’ autonomy by providing trend arrows and optional alerts/alarms to enable pre-emptive management (15), and it can help clinicians and patients evaluate treatment response (dietary measures and pharmacotherapy) using shared metrics, such as postprandial nadir and time below range. Newer CGM systems offer smaller sensors and improved connectivity/alert features; however, fingerstick/capillary or venous glucose confirmation remains mandatory when symptoms occur or when CGM readings are low, particularly during times of rapid glucose change where lag of interstitial glucose and artefacts can lead to false lows (2, 3, 8).

##### Management of incipient hypoglycaemia

Education of patients in identification and management of hypoglycaemia is a key element. In contrast to diabetes hypoglycaemia management, in which ‘rescue’ therapy with high doses of fast-acting carbohydrate is used to correct blood glucose, PBH requires a different and stepwise approach to prevent rebound hyperglycaemia and recurring hypoglycaemia. Patients should be advised to consume small quantities (10–15 g) of fast-acting carbohydrate when hypoglycaemia is confirmed, with a further capillary glucose test after 15 min and re-treatment if still hypoglycaemic, followed by a low GI-carbohydrate snack once euglycaemia has been restored. Sugar in large boluses or sweet drinks is best avoided (11).

##### Lifestyle considerations

Patients should receive counselling on hypoglycaemia recognition and treatment, safety issues (e.g. driving, work, and alcohol), and strategies to reduce exposure to high-risk situations (2, 3). Evidence on exercise timing in PBH remains limited; in contrast to routine advice to avoid activity immediately after meals, a controlled study after RYGB found that a bout of postprandial exercise did not exacerbate hypoglycaemia and may attenuate glycaemic variability in some individuals (25). Activity advice should therefore be individualised (including CGM-guided feedback where available). Stress management and avoidance of unnecessary fasting are also important.

#### Acarbose

Acarbose is an alpha-glucosidase inhibitor that inhibits the degradation and absorption of complex carbohydrates in the small intestine (2). In patients with PBH, acarbose slows carbohydrate absorption, thereby attenuating postprandial glucose and insulin peaks (3, 26, 27). By reducing the magnitude and rate of glucose entry into circulation, it limits both the initial hyperglycaemic peak and the subsequent insulin overshoot that precipitates hypoglycaemia. Although acarbose was originally developed as a glucose-lowering therapy, the rationale for its use in PBH differs from conventional diabetes treatment. In PBH, the aim is to blunt the early postprandial glycaemic peak and thereby reduce downstream incretin-driven insulin hypersecretion, which is thought to contribute to the late postprandial glucose nadir. This approach contrasts with therapies such as somatostatin analogues or GLP-1 receptor antagonists, which more directly attenuate incretin and insulin secretion. Nevertheless, because acarbose reduces intestinal carbohydrate availability, it can theoretically exacerbate hypoglycaemia in the setting of low carbohydrate intake, over-titration, or delayed meals; therefore, cautious dose escalation and glucose monitoring (ideally CGM-supported) are recommended, particularly when used alongside other glucose-blunting agents (2, 17, 27).

Evidence for the efficacy of acarbose in PBH comes from a combination of case reports, small observational series, and controlled studies. Early reports by Moreira *et al.* (33) and Valderas *et al.* (27) described almost complete remission of recurrent hypoglycaemia in post-Roux-en-Y gastric bypass patients treated with acarbose. Subsequent work has reinforced these observations. Ritz *et al.* (26) reviewed the evidence that acarbose blunted both postprandial glucose spikes and insulin responses. Additional studies, such as Frankhouser *et al.* (28), Mordes *et al.* (29), and Cadegiani *et al.* (30), have similarly shown that acarbose substantially reduces glycaemic variability and improves PBH symptom control. Øhrstrøm *et al.* (17) and de Heide *et al.* (31) confirmed these benefits in PBH cohorts. Across studies, patients treated with acarbose typically experience not only fewer symptomatic episodes but also improved day-to-day glycaemic stability. These findings have prompted several expert groups and national guidelines to recommend acarbose as the first-line pharmacological therapy for PBH, particularly when dietary modification alone is insufficient (2, 4, 11). Nevertheless, response to acarbose is variable. While some patients achieve near-complete remission of symptoms, others experience only partial improvement (3, 32). This heterogeneity likely reflects differences in anatomical organisation, beta-cell responsiveness, dietary adherence, and gut hormone dynamics. Importantly, acarbose appears most effective when used in conjunction with structured dietary intervention (4).

In clinical practice, acarbose is taken with meals to target the early absorptive phase of digestion. The typical starting dose is 25 mg with each meal, with gradual titration to 50–100 mg per meal, depending on individual response and tolerability (2, 26). Although gastrointestinal discomfort may limit adherence in a minority of patients, acarbose remains one of the safest and most physiologically rational pharmacologic options for PBH (3). When combined with lifestyle and dietary strategies, acarbose provides an effective, low-risk, and evidence-based first-line therapy that significantly reduces both the frequency and severity of postprandial hypoglycaemia episodes in PBH patients (17, 26, 27, 28, 29, 30, 31, 33).

In addition, patients receiving acarbose must be educated on appropriate hypoglycaemia management. Because acarbose inhibits the breakdown of sucrose, sucrose-containing foods and beverages are ineffective for treating low blood glucose. Instead, patients should use dextrose for correction (as per the approach mentioned above) (2).

#### Surgical management

Surgical options are reserved for a small minority of patients with severe, persistent PBH refractory to structured dietary measures and pharmacotherapy. Reversal of RYGB to normal configuration can delay nutrient transit and dampen exaggerated postprandial hormone responses and may improve hypoglycaemia in carefully selected individuals (31). In addition to complete reversal, surgical revision has been proposed in selected cases – particularly when an anatomical contributor (e.g. partial small bowel obstruction or internal hernia) is suspected (34). However, these approaches are invasive, carry operative risk, and may compromise weight loss benefits; they should be considered only after multidisciplinary evaluation in specialist centres (3).

## Mechanism 2: exaggerated incretin secretion

A hallmark feature of PBH is the exaggerated secretion of incretin hormones, primarily glucagon-like peptide-1 (GLP-1), following nutrient ingestion. After bariatric surgery, the rapid transit of partially digested food into the distal small intestine results in early and direct stimulation of intestinal L-cells, which secrete GLP-1 (35). This amplifies postprandial GLP-1 release (13). GLP-1 acts on pancreatic β-cells to potentiate glucose-stimulated insulin secretion, enhancing both the magnitude and duration of insulin response to meals. In addition, GLP-1 suppresses glucagon release, slows gastric emptying, and promotes satiety, which together modulates postprandial glucose dynamics (36). In PBH, the disproportionate incretin surge leads to excessive insulin secretion relative to the glycaemic load, causing a rapid postprandial decline in glucose levels.

### Treatment options for mechanism 2

#### SGLT inhibitors

Sodium-dependent glucose transporters (SGLTs) play a critical role in glucose transport in humans, with SGLT-1 predominant in the small intestine and SGLT-2 in the renal proximal tubule. Following bariatric surgery, increased SGLT-1 expression in the intestinal mucosa has been associated with enhanced glucose absorption and exaggerated secretion of GLP-1, contributing to postprandial hyperinsulinaemia and hypoglycaemia (2, 4). Inhibiting these transporters therefore provides a physiologically grounded therapeutic strategy for PBH.

Canagliflozin, a dual SGLT-1/2 inhibitor, acts by partially blocking intestinal SGLT-1 and renal SGLT-2. In the intestine, it slows glucose absorption. In the kidney, inhibition of SGLT-2 promotes urinary glucose excretion (37, 38). The combined result is dampening of both hyperglycaemic and hypoglycaemic extremes in PBH. By contrast, empagliflozin is a highly selective SGLT-2 inhibitor that primarily reduces renal glucose reabsorption (18, 39). As a result, dual inhibitors, such as canagliflozin, may offer greater benefits by simultaneously addressing intestinal and renal mechanisms that sustain postprandial glycaemic instability (2, 11).

Evidence supporting SGLT inhibition in PBH is expanding, with canagliflozin demonstrating the most consistent benefit. Abouglila *et al.* (40) and Bellastella *et al.* (41) reported substantial improvements in symptomatic PBH and reduced glycaemic variability in patients treated with canagliflozin. In a prospective interventional study, Ciudin *et al.* (37) demonstrated that canagliflozin effectively blunted postprandial glucose and insulin surges. These results suggest that dual SGLT-1/2 inhibition can simultaneously modulate intestinal absorption and renal glucose reuptake, producing a stabilising effect on postprandial glycaemia. Martinussen *et al.* (38) similarly observed improvements in glycaemic stability in post-bypass patients treated with canagliflozin. Surprisingly, sotagliflozin (another dual SGLT-1/2 inhibitor) has not been evaluated in PBH so far (3).

The findings with empagliflozin are more heterogeneous. Hepprich *et al.* (39) observed reductions in postprandial insulin and improvements in glycaemic variability in PBH patients, whereas Ferreira *et al.* (18), in their randomised, double-blind, placebo-controlled crossover trial in PBH after RYGB, found that empagliflozin 25 mg daily for 20 days reduced glucose excursions but did not reduce CGM time spent (< 3.0 mmol/L). Carpentieri *et al.* (42) attributed this discrepancy to empagliflozin’s renal specificity and lack of intestinal SGLT-1 blockade.

Selective intestinal SGLT-1 inhibition with mizagliflozin is now in active phase 2 evaluation for PBH. A randomised crossover study demonstrated attenuation of postprandial glucose and insulin excursions and fewer hypoglycaemic episodes with single-dose mizagliflozin, with good short-term tolerability (43).

SGLT inhibitors are generally regarded as second-line or adjunctive therapy for patients with PBH who are unable to tolerate acarbose or who require additional glycaemic control despite dietary intervention. They may be particularly beneficial for individuals with concomitant type 2 diabetes, renal diseases, and/or cardiovascular diseases (2, 11). In clinical practice, SGLT inhibition is typically prescribed as a once-daily oral dose (often taken in the morning), consistent with the dosing used in placebo-controlled PBH trials (18). Clinicians should counsel patients regarding hydration, genitourinary infections, and ‘sick-day’ management and should consider renal function and volume status when selecting candidates (3, 4). Because SGLT inhibitors are glucose-lowering drugs, close monitoring (preferably with CGM) is prudent, particularly when used alongside other agents that reduce postprandial glucose excursions (e.g. acarbose), where additive effects could increase the risk of symptomatic hypoglycaemia in some individuals (3, 4, 20).

Overall, current evidence is limited to short-term studies and small sample sizes. Long-term randomised trials are needed to confirm efficacy, safety, and durability and its potential role as combination therapy with other agents, such as acarbose (4, 20). It is also important to acknowledge the apparent paradox of using glucose-lowering agents to treat recurrent hypoglycaemia. Similar to acarbose, SGLT inhibition is used in PBH with the intention of flattening early postprandial glucose excursions that may drive exaggerated GLP-1 and insulin secretion, rather than to lower baseline glucose. However, these agents may still worsen hypoglycaemia in some individuals, particularly with low carbohydrate intake, over-titration, prolonged fasting, or when combined with other therapies that reduce glucose appearance.

#### Somatostatin analogues

Somatostatin analogues, including octreotide and pasireotide, act as synthetic mimetics of somatostatin. They bind to somatostatin receptors expressed on pancreatic β-cells, α-cells, and enteroendocrine L-cells, thereby suppressing the secretion of multiple postprandial hormones, including insulin, glucagon, and GLP-1 (2, 11). By reducing the exaggerated insulin release, these agents blunt the postprandial glycaemic–insulinaemic oscillation and help stabilise blood glucose levels. They also delay intestinal transit and slow gastric emptying, further moderating glucose absorption (46) and perhaps modifying dumping syndrome, which has an overlap with PBH.

Octreotide can reduce late postprandial hypoglycaemia by limiting insulin secretion, but outcomes in post-gastric bypass PBH have been variable across small studies (47, 48, 49). Pasireotide has a broader somatostatin receptor-binding profile than octreotide, which in mechanistic studies can lead to more pronounced suppression of postprandial insulin secretion (11, 51). This broader activity may also increase the risk of transient postprandial hyperglycaemia (46).

Evidence comparing somatostatin analogues in PBH is derived largely from small, short-duration physiological and crossover studies, including studies that evaluated several drugs or doses within the same individuals (17, 46). In mixed meal testing, pre-meal subcutaneous pasireotide (e.g. 75 μg) reduced postprandial GLP-1 and insulin secretion and prevented symptomatic hypoglycaemia in some cohorts (46), extending earlier observations (11, 51). In short studies, pasireotide often produced a flatter postprandial glucose–insulin profile (sometimes with prolonged euglycaemia or hyperglycaemia) and fewer hypoglycaemic events, but these findings are based on limited sample sizes and brief exposure (17, 46, 52). There are no large, long-duration head-to-head trials establishing pasireotide’s superiority over octreotide in routine clinical practice.

Somatostatin analogues are reserved for severe, refractory PBH that does not respond adequately to dietary modification or first-line pharmacotherapies, such as acarbose (2, 50). Both octreotide and pasireotide require subcutaneous or intramuscular administration, often before meals, and their use is limited by side effects, cost, and injection burden.

The most common adverse effects include gastrointestinal disturbances, injection site pain, and gallstone formation due to reduced gallbladder contractility (11). Pasireotide can cause transient postprandial hyperglycaemia through potent insulin suppression, especially at higher doses (150–300 μg) (46).

Patients treated with somatostatin analogues require careful multidisciplinary monitoring, including periodic assessment of glycaemic trends, gallbladder function, and gastrointestinal tolerance. Dose titration should aim for symptom control while avoiding overt hyperglycaemia. Although effective in refractory PBH, the complexity, side effect profile, and cost of these agents currently restrict their use to specialist settings (2, 4).

## Mechanism 3: excessive insulin secretion in response to incretins

An exaggerated insulin response is a central feature of PBH (11, 14). Beyond the enhancement of incretin hormones discussed earlier, several studies suggest that insulin secretion may be inappropriately sustained as glucose levels decline in PBH, contributing to rapid postprandial glucose clearance and subsequent hypoglycaemia (35). Proposed contributors include incretin-driven stimulation, altered autonomic signalling, and adaptive changes in β-cell function; however, the hypothesis of nesidioblastosis or markedly increased β-cell mass in humans remains controversial. Early reports describing islet hyperplasia in highly selected severe cases have not been consistently replicated, and subsequent analyses have suggested no increase in β-cell area/turnover compared with controls, highlighting potential selection bias in pancreatectomy cohorts and uncertainty regarding generalisability (53, 54, 55, 56).

### Treatment options for mechanism 3

#### GLP-1 receptor antagonists

Avexitide (exendin 9–39) is a competitive GLP-1 receptor antagonist that binds to GLP-1 receptors on pancreatic β-cells, blocking the insulinotropic actions of endogenous GLP-1. Patients with PBH exhibit markedly elevated postprandial GLP-1 concentrations, which are thought to drive exaggerated insulin secretion and subsequent hypoglycaemia (14, 35). By preventing GLP-1 from activating its receptor, avexitide reduces excessive insulin release, attenuates the postprandial insulin surge, and thereby stabilises blood glucose levels after meals (11, 57).

In contrast to other PBH treatments that act indirectly on nutrient absorption or pancreatic excitability, avexitide directly targets the incretin–insulin axis, a central mechanism in PBH pathophysiology. GLP-1 receptor blockade leaves glucagon secretion and gastric motility relatively intact, providing a more selective intervention aimed at restoring physiological glucose homeostasis without the broad hormonal inhibition seen with somatostatin analogues (2, 3).

The efficacy of avexitide in PBH is supported by a robust sequence of experimental and clinical studies. Early physiological experiments using intravenous exendin 9–39 demonstrated proof of concept: Salehi *et al.* (35) and Craig *et al.* (58) showed that GLP-1 receptor blockade could nearly abolish post-bypass hypoglycaemia during controlled meal testing, normalising both glucose and insulin excursions. These findings prompted the development of subcutaneous formulations suitable for clinical use.

In a single-dose phase 2 trial, Craig *et al.* (59) reported that a pre-meal subcutaneous injection of avexitide prevented postprandial hypoglycaemia and markedly reduced insulin peaks. A subsequent multiple-dose study established a clear dose–response relationship, confirming that repeated administration maintained euglycaemia across successive meals (57). The pivotal randomised, double-blind, crossover trial conducted by Craig *et al.* (60) evaluated 28 days of daily subcutaneous avexitide treatment in PBH patients. The study demonstrated significant improvements in postprandial glucose profiles, reduced insulin responses, and a substantial decrease in the frequency and severity of symptomatic hypoglycaemic episodes compared with placebo. Importantly, avexitide did not impair fasting glucose levels, indicating that its antagonism was confined to the exaggerated postprandial response.

Avexitide is administered by subcutaneous injection shortly before meals and has shown a favourable short-term safety profile in clinical studies. Reported adverse effects are generally mild and transient, including nausea, headache, and local injection site discomfort (60). Despite encouraging results, avexitide remains an investigational therapy not yet approved for routine clinical use. A phase 3 clinical trial is underway to evaluate its long-term efficacy, durability of response, and safety in a larger PBH cohort (44, 45).

If confirmed effective, avexitide could represent the first disease-specific pharmacological treatment for PBH, addressing the underlying incretin dysregulation (2, 4, 11). However, several practical challenges remain: regular injections are required, long-term cost and availability are uncertain, and the drug’s development pathway has experienced commercial delays following the original developer’s bankruptcy and transfer of rights to another biotechnology company (4).

In addition, MBX-1416, a long-acting GLP-1 receptor antagonist, has reported positive phase 1 results in healthy adults of safety, tolerability, pharmacokinetics, and pharmacodynamics (61).

#### Diazoxide

Diazoxide is an oral ATP-sensitive potassium channel agonist that inhibits insulin secretion by stabilising the open state of β-cell potassium channels. By maintaining membrane hyperpolarisation, diazoxide prevents calcium influx into pancreatic β-cells and thereby reduces insulin exocytosis. This mechanism directly counteracts the exaggerated postprandial insulin release characteristic of PBH, resulting in more stable postprandial glucose levels and fewer hypoglycaemic events.

The drug has been used for decades in hyperinsulinaemic–hypoglycaemic conditions, and although data in PBH remain limited, several small studies and case series suggest potential benefits in selected refractory cases. Spanakis *et al.* (63) first reported marked symptomatic improvements in patients with severe PBH treated with diazoxide, followed by reports by Mathavan *et al.* (64) and Gonzalez *et al.* (65) describing the use of diazoxide in the severe or refractory cases. However, Mordes *et al.* (29) reported no benefit from diazoxide in their study. More recently, de Heide *et al.* (31) documented modest but clinically meaningful improvements in glycaemic stability in a small cohort, while emphasising that diazoxide’s tolerability often limits long-term adherence.

Adverse effects reported in PBH case series include fluid retention/peripheral oedema, nausea, dizziness, and hypotension. Hence, tolerability often limits longer-term use (31, 32). Hypertrichosis/hirsutism is also recognised in other diazoxide-treated patients (e.g. congenital hyperinsulinism), but it has not been a prominent adverse effect in published PBH series. When counselling patients, PBH-specific safety data should be prioritised while acknowledging broader drug class effects, and clinical monitoring for adverse effects is recommended (2).

#### Somatostatin analogues

See the ‘Mechanism 2: exaggerated incretin secretion’ section, above.

## Mechanism 4: impaired alpha-cell glucagon secretion

Whether impaired counter-regulatory glucagon secretion contributes meaningfully to PBH remains debated. Although some studies have reported blunted glucagon responses during hypoglycaemia after RYGB, findings are inconsistent and glucagon abnormalities are unlikely to fully explain the syndrome. Accordingly, exogenous glucagon use in PBH is primarily pragmatic, mirroring its established role in treating severe hypoglycaemia in diabetes, rather than a definitive pathophysiology-targeted therapy (2).

### Treatment options for mechanism 4

In recent years, interest has grown in more physiologically responsive glucagon delivery systems. Studies have investigated mini-dose glucagon and automated delivery approaches that combine real-time CGM data with predictive algorithms to anticipate impending hypoglycaemia, which automatically administer small doses of glucagon to avert symptomatic hypoglycaemia before it develops (66, 67). Clinical feasibility studies in PBH populations have shown that this predictive glucagon infusion model can stabilise glucose profiles, reduce hypoglycaemia frequency, and improve overall glycaemic variability without excessive hyperglycaemic rebound.

Other proof-of-concept studies have evaluated subcutaneous dasiglucagon microdosing using both single-dose meal-test administration and CGM-guided self-administration, demonstrating suppression of postprandial hypoglycaemia (19, 68). Although these data highlight the potential role of glucagon microdosing in PBH, currently available glucagon formulations are only prescribable as relatively large ‘rescue’ doses (e.g. glucagon 1 mg or dasiglucagon 600 μg), which greatly exceed the doses used in experimental microdosing protocols and make them impractical for daily preventive management of PBH. Glucagon microdosing therapy therefore remains experimental, with limited availability outside of research settings and ongoing uncertainties regarding long-term safety, device reliability, and cost-effectiveness.

## Mechanism 5: postprandial 5-HT hypersecretion

Recent studies have identified serotonin (5-hydroxytryptamine, 5-HT) hypersecretion as a potential contributor to the development of PBH. Following RYGB, patients with PBH demonstrated a markedly greater postprandial rise in circulating 5-HT levels (69). This exaggerated 5-HT release from enterochromaffin cells in the gut, and possibly from pancreatic β-cells, appears to potentiate insulin and GLP-1 secretion, thereby amplifying post-meal insulin responses (69, 70). In preclinical models, exogenous serotonin administration has been shown to lower plasma glucose and increase insulin and GLP-1 levels (69).

### Treatment options for mechanism 5

Serotonin receptor antagonism may be a promising therapeutic strategy for PBH. Non-selective 5-HT receptor antagonists, such as cyproheptadine, have already shown potential to blunt serotonin-induced hypoglycaemia in experimental models, while selective 5-HT_2_ receptor blockers (e.g. ketanserin) demonstrate efficacy in normalising postprandial glucose profiles (69). These results suggest that targeted inhibition of 5-HT_2_ receptor signalling might reduce exaggerated insulin release and restore post-meal glucose homeostasis in affected patients. To develop this into practical therapies, future studies should therefore focus on identifying the optimal receptor subtype(s) involved, improving the selectivity and pharmacokinetic properties of antagonists (71).

## Other contributors to PBH

Dysfunctional bile acid signalling could lead to exaggerated fibroblast growth factor-19 (FGF-19) production, affecting the glucose metabolism (2). Changes in the gut microbiome affect nutrient handling and incretin release (4). Inflammatory mediators, such as interleukin-1β, can modulate beta-cell function (3).

## Medications with little supportive evidence for utility in PBH

A few additional pharmacologic agents have been explored in PBH, although the overall evidence remains limited and inconsistent. Calcium channel blockers (CCBs), such as verapamil and nifedipine, inhibit voltage-gated calcium channels on pancreatic β-cells, reducing calcium influx required for insulin exocytosis and thereby moderating postprandial insulin secretion. This theoretical mechanism has prompted off-label use in PBH, aiming to attenuate excessive insulin release and prevent reactive hypoglycaemia. However, available data are restricted to small case reports and observational studies, which have yielded mixed results. Reports by Moreira *et al.* (33), Mathavan *et al.* (64), Vilarrasa *et al.* (32), Ames *et al.* (73), and Øhrstrøm *et al.* (17) described partial or transient symptomatic improvements in some patients, while others experienced minimal benefits.

GLP-1 receptor agonists (GLP-1RAs), including liraglutide and semaglutide, have been suggested as potential therapies for PBH, with hypotheses that sustained receptor stimulation might dampen the exaggerated endogenous GLP-1 secretion or improve glycaemic stability. However, current evidence is limited to small case reports/series and short observational studies with heterogeneous outcomes (62, 72). This approach can appear counterintuitive because GLP-1RAs are glucose-lowering drugs and could theoretically worsen hypoglycaemia in susceptible individuals, reinforcing the need for careful monitoring and properly powered trials. Importantly, GLP-1RAs are increasingly prescribed adjunctively after bariatric surgery for weight regain and cardiometabolic indications; future registry and pharmacoepidemiologic studies may therefore provide indirect evidence regarding their association with PBH frequency and severity in real-world practice.

Another drug of interest is pramlintide, a synthetic analogue of amylin, a peptide hormone co-secreted with insulin by pancreatic β-cells. Pramlintide delays gastric emptying, suppresses postprandial glucagon secretion, and enhances satiety. However, studies in PBH have produced disappointing results. Sheehan *et al.* (74) found no significant improvement in postprandial glucose or hypoglycaemia frequency during MMTT, and some participants developed hypoglycaemia detected on CGM. Pramlintide’s requirement for multiple daily subcutaneous injections and its common gastrointestinal side effects further limit its practicality. As for the newer amylin analogues, such as cagrilintide, these have not been investigated in this context, and their utility is not known.

## Future perspectives

Although recognition of PBH has increased in the past decade, it remains a challenging condition to diagnose and treat. A few areas of research and innovation are opening, which may change management in the years ahead. International consensus on diagnostic criteria, clinically meaningful treatment targets, and standardised outcome measures is warranted. Harmonisation of glucose thresholds (including how CGM metrics are used), symptom endpoints, and reporting standards would facilitate adequately powered multicentre trials and enable comparison across interventions (2, 3, 4).

### Studies on long-term safety and efficacy

One of the key knowledge gaps is the lack of prospective follow-up beyond a few weeks. For a chronic condition such as PBH, future studies should ideally assess durability and safety over at least 6–12 months, including impacts on quality of life, hypoglycaemia awareness, nutritional status, and weight trajectory. Placebo-controlled designs and harmonised endpoints will be important to determine which therapies deliver sustained benefits (2, 3, 4).

### Novel pharmacological targets

The positive outcomes of avexitide (antagonist of GLP-1 receptor) clinical trials have highlighted the potential for targeted therapies. Other novel approaches include the use of 5-HT receptor antagonists, insulin receptor antibodies, glucagon pumps, and bile acid signalling modulators (2, 8, 11, 69). It is hoped that these agents will make a precision approach possible, in contrast to the current off-label use of drugs.

### Combination therapy

Because PBH arises from multiple overlapping mechanisms, combination therapy may be more effective. For instance, combining acarbose with an SGLT inhibitor could theoretically reduce glycaemic variability while minimising side effects (3). However, combining agents that both blunt postprandial glucose appearance (e.g. acarbose plus SGLT inhibition) may also increase the risk of early and/or prolonged hypoglycaemia in susceptible individuals, particularly if carbohydrate intake is reduced or meals are delayed. Combination regimens therefore require careful dose finding, CGM-defined hypoglycaemia safety endpoints, and longer placebo-controlled trials before routine adoption. Similarly, dietary measures combined with GLP-1 receptor antagonism may offer additive benefits. Future trials will be required to establish safety and efficacy of such strategies.

### Innovation in monitoring

CGM technology has evolved considerably and is already altering the landscape of patient management. Newer sensors offer smaller form factors and improved connectivity/alert features; however, accuracy in the hypoglycaemic range remains imperfect, so confirmatory capillary testing is still required when readings are discordant with symptoms. Integration of CGM data with smartphone-based decision support, or even artificial intelligence-driven prediction algorithms, may allow real-time prevention of hypoglycaemia (8, 66, 67), e.g. with glucagon microdosing.

### Biomarkers and personalised care

Another frontier is the identification of biomarkers that predict who will develop PBH and who will respond to specific therapies. Potential candidates include GLP-1 levels, bile acid profiles, and microbiome signatures (4). Such tools could enable risk stratification preoperatively and individualised treatment plans postoperatively.

## Conclusion

PBH represents a complex and heterogeneous complication of bariatric surgery, driven by interrelated disturbances in nutrient transit, incretin signalling, insulin dynamics, and glucagon regulation. While most patients respond to structured dietary strategies that slow carbohydrate absorption and stabilise glycaemic variability, others require targeted pharmacotherapy. Among available options, acarbose remains the best supported first-line agent, acting physiologically to attenuate postprandial glucose excursions. In more severe or refractory cases, SGLT inhibitors and somatostatin analogues offer promising adjunctive or second-line options, while surgical revision is reserved for those unresponsive to medical therapy.

However, it is important to emphasise that, for both pharmacological therapies and surgical interventions, the evidence base is still dominated by small and short-duration studies. As a result, long-term durability and safety data (defined here as ≥6–12 months) remain limited for most interventions. Treatment escalation beyond structured dietary therapy should therefore be individualised and supported by careful monitoring (including CGM where appropriate), with ongoing assessment of nutritional status and weight trajectory, while longer-term prospective and well-controlled studies are undertaken.

Looking ahead, a rational structure for the management of PBH is emerging, with the prospect of tailoring therapy to the dominant pathophysiological mechanisms in each patient. Ongoing research into novel agents, such as 5-HT receptor antagonists, GLP-1 receptor antagonists, and microdose glucagon delivery systems, may soon expand the therapeutic toolkit. Parallel advances in CGM, digital health integration, and biomarker discovery promise to refine diagnosis and enable proactive, data-driven care. Ultimately, multidisciplinary follow-up that integrates endocrinology, dietetics, surgery, and patient education remains essential to mitigate symptoms, improve quality of life, and ensure the long-term metabolic success of bariatric surgery.

## Declaration of interest

TMMT: scientific advisory boards for Amylyx and Nxera; trial steering committee for the PASIPHY phase 2 trial. TMMT and WY: investigators in the PASIPHY phase 2 trial sponsored by Recordati Rare Diseases.

## Funding

This work did not receive any specific grant from any funding agency in the public, commercial, or not-for-profit sector.

## Ethical approval

Not applicable. No new human or animal research was conducted for this review.
